# The importance of early diagnosis and views on newborn screening in metachromatic leukodystrophy: results of a Caregiver Survey in the UK and Republic of Ireland

**DOI:** 10.1186/s13023-022-02550-z

**Published:** 2022-11-03

**Authors:** Georgina Morton, Sophie Thomas, Pat Roberts, Vivienne Clark, Jackie Imrie, Alexandra Morrison

**Affiliations:** 1 ArchAngel MLD Trust, 59 Warwick Square, London, SW1V 2AL, London, UK; 2 MPS Society, MPS House, Amersham, HP7 9LP, Amersham, UK; 3 MLD Support Association UK, Floor 5, Amphenol Business Complex, Thanet way, Whitstable, CT5 3SB, Whitstable, UK; 4 Rare Disease Research Partners (RDRP), MPS House, Amersham, HP7 9LP, Amersham, UK

**Keywords:** Metachromatic leukodystrophy (MLD), Newborn screening (NBS), Inborn errors of metabolism, Diagnostic delay, Gene therapy

## Abstract

Metachromatic Leukodystrophy (MLD) is a rare, autosomal recessive lysosomal storage disorder caused by a deficiency of the enzyme arylsulfatase A (ARSA). MLD causes progressive loss of motor function and severe decline in cognitive function, leading to premature death. Early diagnosis of MLD provides the opportunity to begin treatment before the disease progresses and causes severe disability. MLD is not currently included in newborn screening (NBS) in the UK.

This study consisted of an online survey, and follow-up semi-structured interviews open to MLD patients or caregivers, aged 18 years and over. The aims of the study were to understand the importance of early diagnosis and to establish the views of families and caregivers of patients with MLD on NBS.

A total of 24 patients took part in the survey, representing 20 families (two families had two children with MLD, one family had three children with MLD). Following on from the survey, six parents participated in the interviews. Our data showed diagnostic delay from first symptoms was between 0 and 3 years, with a median of 1 year (n = 18); during this time deterioration was rapid, especially in earlier onset MLD. In patients with late infantile MLD (n = 10), 50% were wheelchair dependent, 30% were unable to speak, and 50% were tube fed when a diagnosis of MLD was confirmed. In patients with early juvenile MLD (n = 5), over half used a wheelchair some of the time, had uncontrollable crying, and difficulty speaking (all 60%) before or at the time of diagnosis. A high degree of support was expressed for NBS among caregivers, 95% described it as very or extremely important and 86% believed detection of MLD at birth would have changed their child’s future. One parent expressed their gratitude for an early diagnosis as a result of familial MLD screening offered at birth and how it had changed their child’s future: “It did and it absolutely has I will be forever grateful for his early diagnosis thanks to his older sister.”

The rapid rate of deterioration in MLD makes it an essential candidate for NBS, particularly now the first gene therapy (Libmeldy™) has been approved by the European Medicines Agency. Libmeldy™ has also been recommended as a treatment option in England and Wales by the National Institute for Health and Care Excellence (NICE) and is being made available to patients in Scotland via the Scottish Medicines Consortium’s ultra-orphan pathway.

## Background

Metachromatic Leukodystrophy (MLD) is a rare, autosomal recessive lysosomal storage disorder caused by a deficiency in the enzyme arylsulfatase A (ARSA) [1, 2], which leads to the accumulation of sulfatides in both the central nervous system and peripheral nervous system [3]. This build-up of storage material causes a progressive loss of gross motor function, severe decline in cognitive function, loss of speech, seizures, muscle spasms, incontinence and ultimately leads to premature death [1].

At present, NBS is offered to every newborn baby in the UK. The heel prick blood spot test is performed when the infant is 5 days old and tests for nine rare and serious health conditions. These are sickle cell disease, cystic fibrosis, congenital hypothyroidism, and inherited metabolic diseases: phenylketonuria (PKU), medium-chain acyl-CoA dehydrogenase deficiency (MCADD), maple syrup urine disease (MSUD), isovaleric acidaemia (IVA), glutaric aciduria type 1 (GA1) and homocystinuria (pyridoxine unresponsive) (HCU) [4]. In some areas of England, NBS for severe combined immunodeficiency (SCID) is also being trialed [4]. Early diagnosis of these disorders within the first weeks of a child’s life provides the opportunity to start treatment at an early age and can prevent disease progression, severe disability or even death, and for some conditions, it allows children access to treatments for which they only qualify at an early age. Currently, MLD is not included in NBS in the UK, and there is a lack of information on the acceptability of NBS from the perspective of those directly affected by the disorder.

The prevalence of MLD is estimated at 1.1 cases per 100,000 live births in the EU [5]. In the UK, the incidence rate is estimated at 1 in 40,000 live births [6]. The clinical phenotype of MLD is due to the global and progressive loss of myelin throughout the nervous system, which leads to a broad range of neurological symptoms [3]. MLD is heterogenous in terms of age of onset, initial symptoms, and progression of symptoms [2]. The exact definition of the different subtypes may vary slightly between sources, although broadly speaking, the most common subtype, late infantile MLD, occurs in the first two years of life [2, 7, 8], and accounts for 40–60% of cases [9]. Children develop symptoms after an initial period of normal development [2]. Symptoms include difficulty walking, loss of speech, muscle weakness and cognitive decline, with the disorder progressing rapidly and death usually occurring between the ages of 5 and 8 years old [6, 9]. Juvenile MLD is often divided into early juvenile and late juvenile forms. Early juvenile MLD accounts for 20–35% of cases [9], children develop symptoms from approximately 3 years of age and disorder progression is less rapid than late infantile [2, 7, 8]. Children develop tremor and muscle rigidity as the disorder progresses, ultimately losing the ability to walk, with death occurring within 10 to 20 years [8, 9]. Late juvenile MLD presents at a later age [2, 7, 8], onset is typically around the age of puberty, with behavioural issues such as aggressiveness, loss of inhibition, lack of judgment and disorientation developing first [2]. The adult subtype is the rarest form of the disorder, and the decline in cognitive abilities may be slow and difficult to recognize [8]. In both the late juvenile and adult forms, cognitive and behavioural issues often prevail before loss of motor function [10].

Diagnosis of MLD is challenging due to the broad spectrum of symptoms and their overlap with other diseases and conditions [2]. MLD is currently diagnosed by biochemical testing using mass spectrometry to quantify sulfatides in dried blood and urine spots [11–13]. Magnetic resonance imaging (MRI) further provides evidence of MLD by a characteristic tigroid pattern in the central white matter [2, 14, 15]. Genetic testing finally establishes the diagnosis of MLD by distinguishing between one of three *ARSA* alleles that result in low ARSA enzyme activity. This is important as both pathogenic *ARSA* variants and *ARSA* variants that cause ARSA pseudodeficiency exist and low residual enzyme activity is not always indicative of MLD [2]. Therefore, to avoid misdiagnosis, a culmination of biochemical, MRI and genetic testing is required to verify a diagnosis of MLD.

Currently, most management is focused on palliative care, although haematopoietic stem cell transplantation (HSCT) is available [7]. HSCT remains controversial and clinical data suggests that it may only be beneficial in the early stages of disease in late-onset patients [16, 17]. A phase 2 trial investigating the efficacy of intrathecal replacement of recombinant ARSA has been completed (Clinical trials identifier: NCT01303146) and a gene therapy (Libmeldy™) for the treatment of late infantile or early juvenile forms, without clinical manifestations of MLD, was approved by the European Medicines Agency in 2020. The US Food and Drug Administration also granted Regenerative Medicine Advanced Therapy (RMAT) designation to Libmeldy™ in 2020 [18]. Libmeldy™ has been recommended as a treatment option by NICE for eligible children in England, Wales and Scotland.

The current study was a collaboration between three patient organisations that support patients and their families with MLD: the Society for Mucopolysaccharide Diseases (MPS Society), the MLD Support Association UK and the ArchAngel MLD Trust. The aims of the study were to understand the importance of early diagnosis by establishing the progression of disease from first symptoms to diagnosis, and to determine the views of families/caregivers of patients with MLD on newborn screening for this disorder.

## Methods

### Recruitment

Members of three patient organisations, the MPS Society, the MLD Support Association UK and the ArchAngel MLD Trust were invited by email and telephone to participate. To be eligible, parents/carergivers/patients had to be aged ≥ 18 years and resident of the UK or Republic of Ireland. Participants had to be the parent or caregiver of a live or deceased person with a confirmed diagnosis of MLD and be able to provide informed consent to participate. Parents or a caregiver with more than one child with MLD, were asked to complete a separate questionnaire (and interview, if applicable) for each child. The results reported here are part of a larger study, examining the burden of disease and the patient and caregiver experience in MLD, that will be reported elsewhere.

### Survey questionnaire and interviews

The online survey used a specifically designed questionnaire covering demographics, diagnosis, symptoms and disease progression, burden of illness, treatment, and NBS. Consent was sought at the start of the online survey and additional consent was sought for participation in the follow-up interviews. Questions were presented as multiple choice where possible, with free text to include additional information not covered by the answer options. The online surveys were completed between 28 August and 18 October 2020.

Respondents who had completed the online survey were eligible to take part in the in-depth interviews. A semi-structured interview guide was developed by patient organisations in collaboration with RDRP, designed to explore further the items raised in the online survey. Interviews were all conducted over the telephone with the same member of the MPS Society’s patient services team and took place between 29 September and 21 October 2020. Responses were analysed applying an inductive thematic content approach and using the computer Qualitative Data Analysis (QDA) software NVivo. Data were aggregated and remained anonymous and no personally identifiable data were collected. Participants could decline to answer any question and were able to stop the interview at any point. Permission specifically to use quotes from the recordings was sought, and participants could indicate any content that could not be cited. Analysis of the online survey results and interview transcripts was undertaken by RDRP. This research was conducted in accordance with the British Healthcare Business Intelligence Association’s Legal & Ethical Guidelines for Market Research [19].

### Disease progression

In the online survey, respondents were asked to indicate the presence or absence of symptoms at various time points to gain an understanding of the progression of MLD. Time points used to capture symptoms included: first symptoms, at time of diagnosis, current symptoms or in the final stage of disease if deceased. Symptoms were presented as multiple-choice lists under the following headings with options to add other symptoms as free text: mobility; skeleton, muscles, joints; eyesight and hearing; behaviour; learning and understanding (cognitive); neurological; speech and communication; nutrition and eating; chest and respiratory, and bowels and bladder. The rate of progression was explored further in the interviews.

The definitions used for the MLD clinical subtypes were:

- Late infantile (symptom onset ≤ 2.5 years of age).
- Early juvenile (symptom onset > 2.5 to < 7 years of age).
- Late juvenile (symptom onset 7 to < 17 years of age).
- Adult onset (symptom onset ≥ 17 years of age).

### NBS

In the online survey, respondents were asked for their views on NBS. Respondents were asked questions on information they had received on current NBS tests, availability, interpretation, and outcome of results. Respondents were also asked what the effect of a positive result would have on their future reproductive choices and if they would be willing to support an application for MLD to be added to the UK NBS programme.

## Results

### Patient demographics

A total of 24 patients were included in the study, representing 20 families. This represents around half of all patients known to the patient organisations. Respondents were mostly parents of patients that were alive at the time of the survey (n = 21), with the remaining comprising bereaved parents (n = 2) and bereaved carergivers (n = 1). The median age of patients was 7.3 years, and three patients were deceased. One third (n = 8) of patients had a sibling with a confirmed diagnosis of MLD. Of the patients in the study, thirteen had late infantile MLD, six had early juvenile MLD, two had late juvenile MLD and three had adult onset MLD. In total, 58% (n = 14) were female and 88% (n = 21) were from England, with the remaining patients from Republic of Ireland (8%, n = 2) and Northern Ireland (4%, n = 1).

### Diagnostic delay and disease progression

Diagnostic delay was defined as time from the first symptom to diagnosis of MLD. This was a qualitative measure reported by the parent or caregiver. Six patients were excluded from the analysis of diagnostic delay, of these, four patients were diagnosed before symptoms due to the diagnosis of an older sibling and two patients failed to provide the age at which first symptoms appeared. The median age of patients when symptoms first appeared was 2.8 years, and the median age of patients when diagnosed was 4.3 years (Table 1). Diagnostic delay was between 0 and 3 years, with a median of 1 year (n = 18), during this time deterioration was rapid, especially in earlier onset MLD (Table 2).

**Table 1:** Patient characteristics

	Late infantile (N = 13)	Early juvenile (N = 6)	Late juvenile (N = 2)	Adult onset (N = 3)	Total (N = 24)
**Current age or age at death**^**a**^, **years**					
Mean (SD)	5.5 (1.5)	12.3 (2.3)	24.0 (12.7)	39.0 (9.0)	12.9 (12.1)
Median	6 0.0	12.5	24.0	39.0	7.3
Range	2–7.5	9–16	15–33	30–48	2–48
**Age symptoms first appeared, years**					
Mean (SD)	1.4 (0.7)^b^	4.6 (1.1)^c^	10.5 (4.9)	22.5 (3.5)^d^	5.7 (7.0)^e^
Median	1.3^b^	5.0^c^	10.5	22.5^d^	2.8^e^
Range	0.3–2.5^b^	3–6^c^	7–14	20–25^d^	0.3–25^e^
**Age at diagnosis, years**					
Mean (SD)	2.4 (0.4)^b^	5.9 (0.3)^c^	11.5 (4.9)	24.5 (2.1)^d^	6.9 (7.2)^e^
Median	2.5^b^	6.0^c^	11.5	24.5^d^	4.3^e^
Range	1.5–3^b^	5.5–6.2^c^	8–15	23–26^d^	1.5–26^e^
**Diagnostic delay, years**					
Mean (SD)	0.9 (0.8)^b^	1.3 (1.2)^c^	1.0 (0)	2.0 (1.4)^d^	1.2 (0.9)^e^
Median	0.8^b^	1.2^c^	1.0	2.0^d^	1.0^e^
Range	0–2.3^b^	0–3^c^	0	1–3^d^	0–3^e^

^a^ Three patients were deceased at the time of the survey

^b^ Reported for 9 patients only

^c^ Reported for 5 patients only

^d^ Reported for 2 patients only

^e^ Reported for 18 patients only

SD = standard deviation

**Table 2:** First symptoms and symptoms at diagnosis (mobility, speech, swallow, and continence)

Patients, n (%)	Late infantile (N = 10)		Early juvenile (N = 5)		Late juvenile (N = 2)		Adult onset (N = 3)	
	**First symptoms**	**Symptoms at diagnosis**	**First symptoms**	**Symptoms at diagnosis**	**First symptoms**	**Symptoms at diagnosis**	**First symptoms**	**Symptoms at diagnosis**
*Mobility* Some issues with walking Losing ability to walk Use of wheelchair some of the time Wheelchair dependent None of the above symptoms	4 (40) 5 (50) - - 1 (10)	1 (10)^a^ 2 (20)^a^ 1 (10) 5 (50) 1 (10)	4 (80) 1 (20) - - -	1 (20) 1 (20) 3 (60) - -	1 (50) - - - 1 (50)	- 2 (100) - - -	- - - - -	- 1 (33) - - 2 (67)
*Speech and communication* Lost ability to speak Lost ability to articulate pain or discomfort Lost ability for non-verbal communication Difficulties with speech (dysphasia, dysarthria, speech deteriorating) None of the above symptoms	- - - 3 (30) 7 (70)	3 (30) 3 (30) 2 (20) 4 (40) 3 (30)	- - - - 5 (100)	- - - 3 (60) 2 (40)	- - - - 2 (100)	- - - - 2 (100)	- - - 1 (33) 2 (67)	1 (33) 1 (33) 1 (33) 1 (33) 1 (33)
*Swallow and feeding* Difficulty swallowing/risk of choking Fed by nasogastric tube Fed via gastrostomy tube None of the above symptoms	4 (40) 1 (10) - 5 (50)	1 (10)^a^ 4 (40)^a^ 1 (10) 4 (40)	- - - 5 (100)	- 1 (20) - 4 (80)	- - - 2 (100)	- - - 2 (100)	- - - 3 (100)	1 (33) - - 2 (67)
*Continence* Urgency/frequent accidents Urinary incontinence Bowel incontinence Double incontinence None of the above symptoms	- - - 1 (10) 9 (10)	- - 1 (10) 3 (30) 6 (10)	2 (40) 1 (20) - - 2 (40)	1 (20) 1 (20) - 1 (20) 2 (40)	- - - - 2 (100)	- - - - 2 (100)	- - - - 3 (100)	- 1 (33) - - 2 (67)

^a^ Includes one respondent who provided no answer for symptom at diagnosis, therefore assumed symptom reported as first symptom was also present at diagnosis

### Late infantile MLD

Three patients with late infantile MLD were diagnosed before symptoms appeared due to diagnosis of an older sibling, and inconsistent answers regarding age at symptom onset and diagnosis were reported for one patient. These four patients were therefore excluded from the analysis of diagnostic delay. The median age of patients when symptoms first appeared was 1.3 years (n = 9). The median age of patients when diagnosed was 2.5 years (n = 9, Table 1). Diagnostic delay was between 0 and 2.3 years, with a median of 0.8 years (n = 9, Table 1), and deterioration was rapid (Table 3, Late infantile).

**Table 3:** Case studies: pathways to diagnosis

MLD subtype	Diagnostic journey
Late infantile: Case study 1	• First symptoms were observed at 2 years old. • The child did not progress from walking holding on to things to walking independently. • The child saw various doctors and hip dysplasia was suspected. During this time the child was finding it more difficult to walk and was referred to a ***community paediatrician*** who sent for blood tests and MRI. • Late infantile MLD was diagnosed at 2 years and 8 months old. **Interviewer**: *“So, between the first symptoms and when you got the diagnosis, had your daughter deteriorated any further?”* **Parent**: *“Yes. She had deteriorated more by that point. Her speech had slurred quite significantly, and she was dribbling excessively. And her sleep was really disturbed as well.”*
Late infantile: Case study 2	• First symptoms were observed at 1 year old. • The child did not progress from walking holding on to things to walking independently. • The child saw various doctors and parents noticed a decline at 18 months old. • First referred to a ***community physiotherapist*** for walking issues, child was getting worse, after a long time was referred to a ***paediatrician***. • The child was misdiagnosed with hyperkalaemia and neuropathy. • Genetic tests and initial MRI were inconclusive. • MLD was suspected after the second MRI and genetic tests confirmed diagnosis three months later. • Late infantile MLD was diagnosed at 2 years and 6 months old. **Interviewer**: *“What led them to do the MRI?”* **Parent**: *“We knew that he was getting worse, and we had always kind of had to speak up for [name] and had some disagreements with the team and what they thought, and we demanded that he be seen again, that he was regressing. And so, they did the MRI the second day and they discovered that there was white matter accumulating in the brain and in that respect when they looked at the first MRI, they also discovered that they should have seen it back then too. 18 months lost.”*
Late infantile: Case study 3	• First symptoms were observed at 3 months old. • Parents were concerned that the child had a degenerative condition from an early age. • The child first went to the ***GP*** with feeding issues where they saw a ***breastfeeding specialist*** who noted the child had an asymmetric jaw. • Numerous visits to the GP for frequent chest infections, concerns over breathing at night and floppy baby were recorded. • This led to referral to ***paediatrician 1***, who felt that feeding issues were due to reflux. • Further visits to the GP were made, the child was very ill for 6 weeks, sick at every feed, and had a temperature. • The child did not pass urine for 24 h and was then hospitalised with pneumonia. The parents asked for help as they felt that the first paediatrician was not listening to them. • This led to referral to ***paediatrician 2***, who referred the child to physiotherapy. • The ***physiotherapist*** made some progress, but the child then started to regress. • The ***midwife*** noted delayed growth and motor skills at the “2-year check”. Referred the child to a community paediatrician. • The parents of the child talked through all their concerns with the ***community paediatrician***, assessments were done, and the parents were told child was just delayed. • The mother persevered and asked for a test for muscular dystrophy, the child was referred to a neurologist. • The ***neurologist*** was concerned, tests were conducted, and a diagnosis was reached. • Late infantile MLD was diagnosed at 2 years and 6 months old. **Parent**: *“We got rushed into the hospital and that’s where I met the second consultant, where I just broke down and said, I know he’s got a chest infection, but I think there’s more than this. I think there’s more to it than this and I feel like nobody’s listening to me. Like the doctor’s not listened to me, the other paediatrician didn’t listen to me. And I feel like we just need some help.”*
Early juvenile: Case study 1	• First symptoms were observed at 5 years old. • The child had previously been bright, but had lost interest in reading, was becoming clumsy and had wet themselves a few times. Behaviour issues were also reported at school. • The ***GP*** thought the child might be having petit mal seizures. • The child’s nursery teacher offered to assess them and could see there had been a significant change – she spoke to the doctor. • The doctor referred the child for a CT scan and a brain degenerative condition was confirmed. • The child had an MRI in July and by September was unable to walk. • Early juvenile MLD was diagnosed at 5 years and 6 months old. **Parent**: *“I was quite often just shrugged off as a neurotic mother, I think. There was various things that just weren’t adding up to me. Just little things. And our initial thoughts were that she wasn’t settling in very well for school. She’d just started reception. And I had approached the school for help many times weekly. And probably on a weekly basis, I was in asking for her to be referred somewhere. And I was just constantly met with… Just made to be obviously neurotic, really. And she was just a naughty, difficult child.”*
Early juvenile: Case study 2	• First symptoms were observed at 3 years old. • The child started tripping up. • By age 4, the child would get frustrated trying to pull up a zip or put a lid on a pen. • The ***GP*** reassured the parents that children just develop at different rates and by age 5 all children have caught up. • The parents went back to the GP with more symptoms, which were getting worse, including constant frustration and behavioural issues. GP referred child to a ***psychologist*** and a ***child development unit***. • A series of assessments were done, the school noted that the child’s hands would shake when they picked up a pen. Dyspraxia was diagnosed and ***occupational therapy*** given. • Parents were concerned about the hand tremor and had researched it and felt there could be a neurological issue. The GP referred them back to the child development unit. • The child development unit were resistant but agreed to do an MRI and blood tests. Some underdevelopment in myelination was found but they were told this was nothing to worry about. • The parents pushed for further investigation and MRI was sent to ***neurologist*** for review towards the end of the year. • Parents could not get in touch with the ***paediatrician*** to find out results, calls and emails were not answered. Diagnosis was finally given; paediatrician did not know about the disease and suggested the parents research it. • Parents found out that it was metabolic and approached ***Great Ormond Street Hospital*** where confirmatory diagnostic testing was done. • Early juvenile MLD was diagnosed at 6 years old.
Adult onset: Case study 1	• First symptoms were observed at 20–21 years old. • The patient was at university and had become forgetful. However, in hindsight with an understanding of MLD, there were some early signs from 17 years old. The patient achieved lower grades than expected in A-levels and showed signs of aggression. • The patient was referred to a ***psychiatrist*** and investigated for schizophrenia and other possible causes. The trigger for diagnosis was when the patient could no longer tell the time. • The Parents pushed for further investigation and an MRI was done. • Adult onset MLD was diagnosed at 23 years old. **Parent**: *“If we’d got the diagnosis a year earlier, he would probably have been living independent life still, albeit supported. Because it was that last year, was really when the symptoms started to manifest. And it was obvious we couldn’t leave him alone for any length of time. We had to monitor what was happening. He’d put a meal in the oven to cook and then go out.”*

While most children had met their early developmental milestones for speech and learning around two thirds had not achieved their walking milestones. The most common first symptoms included issues with walking (n = 9, Table 2), difficulty swallowing (n = 4, Table 2), hypotonia (n = 5, Table 4), and hypertonia (n = 4, Table 4). One parent described the many issues that were present from an early age:

**Table 4:** Other first symptoms and symptoms present before diagnosis (including first symptoms)

Patients, n (%)	Late infantile (N = 10)		Early juvenile (N = 5)		Late juvenile (N = 2)		Adult onset (N = 3)	
	**First symptoms**	**Symptoms before diagnosis**	**First symptoms**	**Symptoms before diagnosis**	**First symptoms**	**Symptoms before diagnosis**	**First symptoms**	**Symptoms before diagnosis**
*Skeleton, muscles, or joints* Dystonia Hypotonia Hip subluxation Scoliosis Spasticity or hypertonia None of the above symptoms	1 (10) 5 (50) - - 4 (40) 3 (30)	5 (50) 6 (60) - - 6 (60) 3 (30)	- 1 (20) - - 1 (20) 4 (80)	- 1 (20) - - 1 (20) 4 (80)	- - - - 1 (50) 1 (50)	- - - - 1 (50) 1 (50)	- - - - - 3 (100)	1 (33) 1 (33) 1 (33) 1 (33) 1 (33) 2 (67)
*Eyesight* Vision difficulties (glasses needed) Blindness None of the above symptoms	2 (20) - 8 (80)	5 (50) - 5 (50)	- - 5 (100)	- - 5 (100)	- - 2 (100)	- - 2 (100)	- - 3 (100)	- 1 (100) 2 (67)
*Hearing* Hearing difficulties (hearing aid needed) Deafness None of the above symptoms	- - 10 (100)	- - 10 (100)	- - 5 (100)	- - 5 (100)	- - 2 (100)	- - 2 (100)	- - 3 (100)	- 1 (100) 2 (67)
*Behavioural symptoms* Challenging or difficult behaviour Hyperactivity and/or repetitive behaviour No awareness of danger Change in personality None of the above symptoms	- - - - 10 (100)	- - - - 10 (100)	3 (60) 2 (40) 3 (60) 3 (60) 1 (20)	3 (60) 2 (40) 3 (60) 3 (60) 1 (20)	- - - - 2 (100)	- - - - 2 (100)	2 (67) 1 (33) 2 (67) 3 (100) -	3 (100) 1 (33) 2 (67) 3 (100) -
*Cognitive symptoms* Confusion or disorientation Learning issues Memory and concentration issues Dementia None of the above symptoms	- 2 (20) 1 (10) - 8 (80)	1 (10) 3 (30) 4 (40) - 6 (60)	1 (20) 3 (60) 3 (60) - 1 (20)	1 (20) 5 (100) 3 (60) - -	- 1 (50) - - 1 (50)	- 2 (100) 1 (50) - -	2 (67) 2 (67) 3 (100) 1 (33) -	2 (67) 2 (67) 3 (100) 2 (67) -
*Neurological symptoms* Anxiety or panic Issues with temperature regulation Peripheral neuropathy Seizures/epilepsy Sensory processing issues Sleep disturbance Uncontrollable crying None of the above symptoms	1 (10) 1 (10) 2 (20) 1 (10) 1 (10) 1 (10) 2 (20) 6 (60)	5 (50) 3 (30) 2 (20) 2 (20) 3 (30) 4 (40) 4 (40) 3 (30)	1 (20) 1 (20) 1 (20) - 1 (20) 2 (40) 2 (40) 1 (20)	1 (20) 2 (40) 2 (40) - 1 (20) 2 (40) 3 (60) -	- - - - - - - 2 (100)	- - - - - 1 (50) - 1 (50)	- - - - - 1 (33) - 2 (67)	1 (33) 1 (33) 1 (33) 2 (67) 1 (33) 2 (67) - 1 (33)
*Gallbladder issues* Gallbladder issues	-	2 (20)	-	1 (20)	-	-	-	1 (33)
*Chest and respiratory symptoms* Aspiration Excess secretions Frequent chest infections Frequent colds or runny nose None of the above symptoms	1 (10) 3 (30) 1 (10) 3 (30) 6 (60)	3 (30) 4 (40) 2 (20) 3 (30) 6 (60)	- - - - 5 (100)	- - - - 5 (100)	- - - - 2 (100)	- - - - 2 (100)	- - - - 3 (100)	- - - - 3 (100)

*As a baby [Name] was floppy, breathing was a concern and had a poor suck and swallow making feeding hard. This was suggested to be due to an asymmetric jaw. He had a small fontanelle, which is what got us the initial appointment with the paediatrician. His head size was a concern and his fingers didn’t always open. As time went on [Name] had pronated feet, making it difficult to stand and this opened up the door to physiotherapy, before he was two. [Name] struggled to eat food, this was a long journey right from the beginning and alongside this speech was delayed.*

Another parent described how issues with walking had been one of the first signs that something was wrong:

*She’d started to walk, but she wouldn’t progress from walking holding onto things to walking independently. So, when she got to two years old, that’s when we first went to the doctors, thinking that maybe something wasn’t quite right, because she just wasn’t going past that next stage.*

Rapid disease progression was seen in the time taken to reach diagnosis. By the time of diagnosis, 50% (5/10) were wheelchair dependent, 30% were unable to speak, and 50% were tube fed (Table 2). One parent describes how their child went from crawling up and down stairs to being completely bedbound over a period of six months from diagnosis:

*…we noticed that her walking even when holding onto things was then becoming more difficult for her. [By the time of diagnosis] she had deteriorated more. Her speech had slurred quite significantly, and she was dribbling excessively. And her sleep was really disturbed as well. She would take a long time to fall asleep and she would cry a lot as well. She was in pain, but it wasn’t obvious where she might be in pain.…it wasn’t long after her diagnosis that she had her first seizure and we had the ambulance out.*

Mobility issues were followed by a rapid decline in speech and cognition for another child in a 9–12-month period, between the age of 2–3 years. As physical decline occurred before cognitive decline, the rapid loss of skills was particularly distressing for the child and parents. The child had deteriorated from being able to eat and drink independently, to total dependence on their parents. By the time of diagnosis, he was being fed by a naso-gastric tube and needed a gastrostomy at age 3 years.

*…he was speaking very well compared to others his age. He was very talkative and fine at two. At that age then he began to slow down speaking and slowly but surely losing all his ability. He was really quite well developed at two years of age as a little boy but over the course of about nine months all that disappeared on him. From that age of two to three where he lost his physical ability before his mental faculties was very traumatic for him and for us and physically painful and emotionally upsetting and confusing and distressing for [Name] and for us. It was terrible to watch him.*

Another parent described the loss of skills that occurred before her child was diagnosed with MLD. The child had been pulling themselves up to stand, was crawling, and was quick at going upstairs. By the age of 2, the parent started to notice a decline and the child started using a walker. In less than a year, the child had stopped crawling, walking, speaking and eating.

*Like he didn’t go up the stairs as quick as he did. He wasn’t pulling himself to stand as much. He was sitting more. And if he did there was a bit more reluctance there.”* The child had an MRI and was diagnosed with MLD 6 months later. *“He started showing less interest in doing things. And then by the time we came round to the MRI scan, he’d already not been going up the stairs. He’d already not been pulling himself up to stand. He was still crawling, and he crawled most of that year.…and then by the Christmas he wasn’t doing any of it. So no crawling, no talking, no walking, no eating. We had three months and it all just went really quickly.*

In most cases, the diagnostic journey was long, with multiple referrals, doctors, and specialists required to eventually confirm MLD disease, often referred to as “diagnostic odyssey”. In two patients, the deterioration between the first symptoms and diagnosis was extremely apparent (Table 3, Late infantile: case study 1 and 2). In the case of one child, first symptoms were observed at 3 months old, and the parents were concerned that the child had a degenerative condition from an early age. The child was seen multiple times by the GP with feeding issues, chest infections and concerns over breathing at night. The child was referred to a paediatrician who according to the parents, ultimately disregarded their concerns. Subsequent visits to the GP and hospitalisation led to a second paediatrician referral:

*We got rushed into the hospital and that’s where I met the second consultant, where I just broke down and said, I know he’s got a chest infection, but I think there’s more than this. I think there’s more to it than this and I feel like nobody’s listening to me. Like the doctor’s not listened to me, the other paediatrician didn’t listen to me. And I feel like we just need some help.*

The child was seen by a physiotherapist and then a midwife, who referred the child to a community paediatrician. Assessments were carried out and the parents were told that the child was just delayed. The mother persevered, and the child was referred to a neurologist. Finally, late infantile MLD was diagnosed at 2 years and 6 months (Table 3 Late infantile: case study 3).

### Early juvenile MLD

One child with early juvenile MLD was diagnosed before symptoms appeared due to diagnosis of an older sibling and were excluded from the analysis of diagnostic delay leaving a total of five children who were symptomatic before diagnosis. The median age of patients when symptoms first appeared was 5.0 years (n = 5), and the median age of patients when diagnosed was 6.0 years (n = 5, Table 1). Diagnostic delay was between 0 and 3 years, with a median of 1.2 years (n = 5) and during this time deterioration was rapid (Table 3, Early juvenile).

All children had met their early developmental milestones for speech, learning, and walking. Initial symptoms included issues with walking, toileting, and learning/behavioural problems (Tables 2 and 4). At diagnosis, 60% (n = 3) were starting to use a wheelchair, 60% (n = 3) had difficulty speaking (Table 2), and 60% (n = 4) had uncontrollable crying (Table 4). One parent described the rapid progression from first symptoms that were noticed when the child started pre-school to diagnosis six months later:

*Yes, certainly she started becoming more clumsy, I would say. But even up until the MRI, which happened about two weeks after the computerized tomography (CT) scan, she was still walking normally. Although maybe slightly heavier on her feet, almost like a flat-footed type sound she was making. But then after she’d had the MRI, she almost… She just deteriorated quite rapidly after the MRI. But I would say maybe a week to two weeks after the MRI, you could really see that she was struggling with overall walking. She was still walking, but she was leaning forward for balance reasons, I guess. And she couldn’t run for any length of time either. It was very quick, within a couple of months. Certainly, within three months of having a CT scan, she’d stopped walking.*

In one case, the first symptoms appeared at 5 years old. The child had previously been bright but had lost interest in reading, become clumsy, and had wet themselves a few times. The school had also reported behaviour issues. The mother of the child recounted how she felt:

*I was quite often just shrugged off as a neurotic mother, I think. There was various things that just weren’t adding up to me. Just little things. And our initial thoughts were that she wasn’t settling in very well for school. She’d just started reception. And I had approached the school for help many times weekly. And probably on a weekly basis, I was in asking for her to be referred somewhere. And I was just constantly met with… Just made to be obviously neurotic, really. And she was just a naughty, difficult child.*

After the child’s nursery teacher spoke to the doctor, the child had a CT scan, and a brain degenerative condition was confirmed. The child had an MRI in July and by September was unable to walk. Early juvenile MLD was diagnosed at 5 years and 6 months old (Table 3, Early juvenile: case study 1). In another case, the first symptoms appeared at 3 years old when the child began to fall over. A decline in motor function and issues with behaviour followed and after much perseverance from the parents to achieve a diagnosis, early juvenile MLD was finally diagnosed 3 years later (Table 3, Early juvenile: case study 2).

### Late juvenile MLD

The two patients were symptomatic before diagnosis and some disease progression was observed in this period. The median age of patients when symptoms first appeared was 10.5 years and the median age of patients when diagnosed was 11.5 years, with a median diagnostic delay of 1 year (n = 2, Table 1). In late juvenile MLD, both patients had met all their early developmental milestones. Initially, 50% (n = 1) reported issues with walking (Table 2), 50% (n = 1) presented with hypertonia, and 50% (n = 1) had learning difficulties (Table 4). At diagnosis, both patients had started to lose the ability to walk and had learning issues. One patient had hypertonia, and one patient had memory and concentration issues (Tables 2 and 4).

### Adult onset MLD

All three patients were symptomatic before diagnosis, however, age at first symptoms, diagnosis, and subsequent delay in diagnosis were only recorded for two patients. The median age of patients when symptoms first appeared was 22.5 years, and the median age of patients when diagnosed was 24.5 years, with a median diagnostic delay of 2 years (Table 1).

One patient had not met their early developmental milestones, initial symptoms were a change in behaviour and cognitive deterioration (Table 4). In the case of one patient with adult onset MLD, the first symptoms were observed at 20–21 years old. The parent described how the first symptoms were appearing while their child was at university. Doctors initially thought the problem was psychiatric and it was not until the patient lost the ability to tell the time that further tests were done.

*I think the difficulty we had; he was away at university. And he was able to mask a lot of the symptoms. So, I suspect the symptoms had actually started a lot earlier. But we first really noticed when [patient] was about 20, 21 that he was becoming forgetful and that. And that’s really when we took him to the doctors, and we finally, finally got a diagnosis when he was 23 after being lumped into psychiatry. No, what really led to [diagnosis] was when he suddenly stopped being able to tell the time. I finally managed to get him out of the hands of the psychiatrist and get an MRI scan done.*

The parents strongly believed that an earlier diagnosis would have led to a far brighter future for their son:

*If we’d got the diagnosis a year earlier, he would probably have been living independent life still, albeit supported. Because it was that last year, was really when the symptoms started to manifest. And it was obvious we couldn’t leave him alone for any length of time. We had to monitor what was happening. He’d put a meal in the oven to cook and then go out.*

Adult onset MLD was diagnosed at 23 years old.

### NBS: family views

**Fig. 1:**
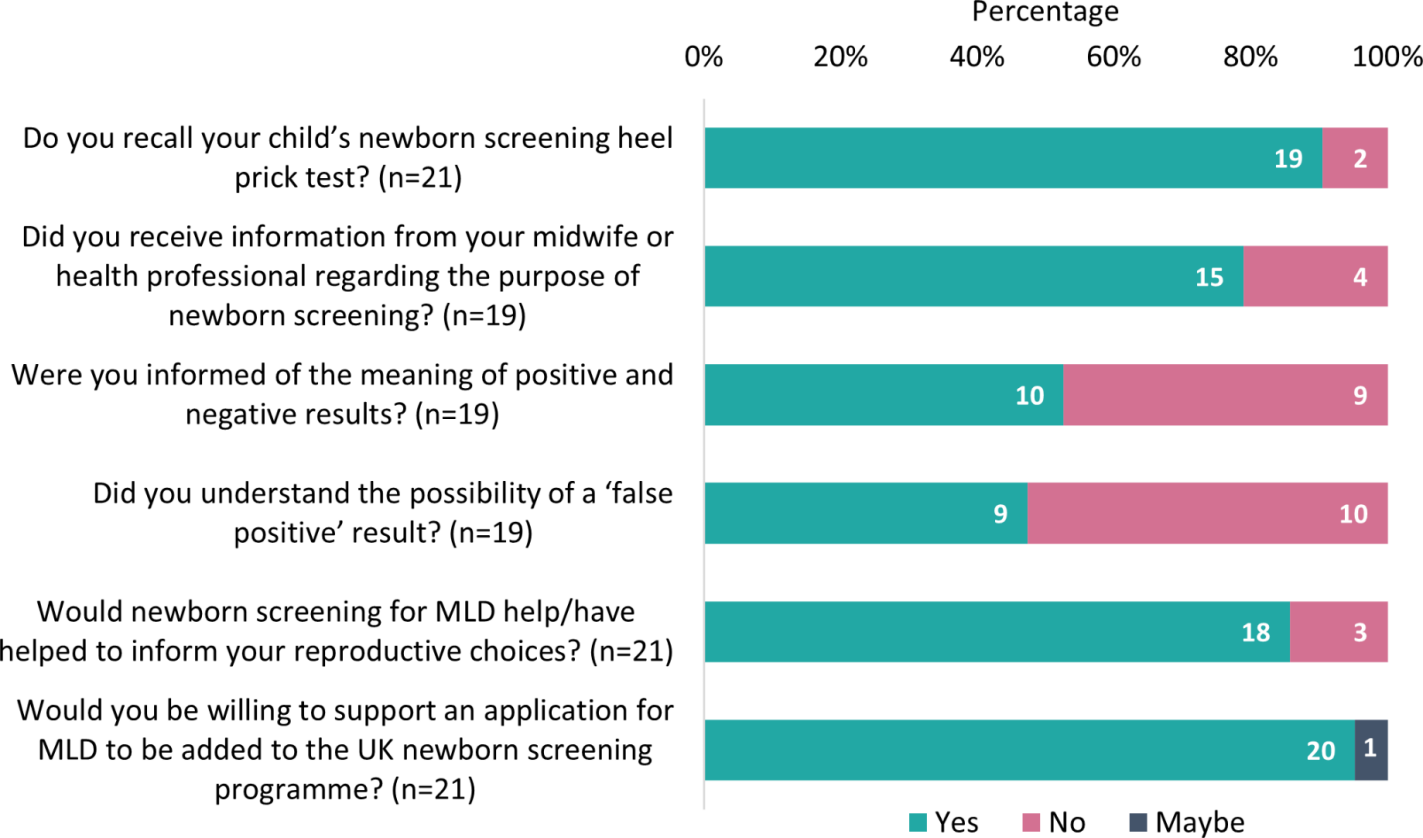
Family views on NBS: results from the online survey

Responses were received from all 20 families taking part in the survey. In one family, both the father and mother replied, giving 21 responses in total. The questions and responses are summarised in Fig. 1.

### Information about NBS and the heel prick test

The majority (79%) of parents received information from healthcare professionals about the purpose of NBS. Only 2 parents (10%) were not able to recall their child’s NBS heel prick test.

### Interpretation of screening results

Approximately half of respondents were informed of the meaning of positive and negative results (53%), and just under half understood the possibility of obtaining a “false positive” result (47%).

### Outcome of NBS results and effect upon reproductive choices

Most respondents (80%) considered an undetected case of MLD at birth as more harmful than a false positive screening result. When respondents were asked if NBS for MLD would have helped to inform their reproductive choices, 86% said that it would have helped to make an educated decision, whilst the remaining respondents said that it would not have affected their reproductive choices or they were too old by the time of diagnosis. The majority of respondents (86%) believed detection at birth would have changed their child’s future. One parent described the torment of realising they were too late for medical intervention:

*Yes, [child] had no symptoms pre diagnosis. She did have dyslexia but so do I and her sister and cousin. [She] woke up one morning saying she could not walk. She had a scan 2hrs later then a blood test to confirm scan diagnosis. We was shell shocked and at the time not very good at researching. The doctors did not tell us about trials. We have to live with knowing we was too late. A test at birth could of changed that.*

Another respondent expressed their gratitude for an early diagnosis as a result of familial MLD screening offered at birth and how it had changed their child’s future:

*It did and it absolutely has I will be forever grateful for his early diagnosis thanks to his older sister.*

Three respondents with offspring who were diagnosed with adult onset MLD thought that detecting MLD at birth would not have changed their child’s future. One respondent said:

*No because they lived a good life, went to school, got jobs, married and had families.*

One parent felt that NBS for MLD would not have influenced their child’s future as treatments were not yet available:

*Probably not as treatments to delay or prevent symptoms were not available until after his condition was already significantly degenerated.*

### Support for NBS

Overall, there was a high degree of support for NBS among caregivers, with 95% describing it as very or extremely important and 5% describing it as not at all important. Twenty out of 21 respondents were willing to support an application for MLD to be added to the UK NBS programme.

## Discussion

Due to the rarity and severity of the disease, limited data on patients with MLD are available. Our qualitative study involving parents and caregivers of patients with MLD, collected information on first symptoms, age of diagnosis and views on NBS. The variability of symptoms in MLD, coupled with the very low incidence rate, often mean that the disease is misdiagnosed or diagnosed too late for patients to be considered for treatment [8]. Our survey and interviews revealed that it can take up to three years from the first symptoms to diagnosis and were similar to those reported in a recent study, which reported a mean time from first symptom to diagnosis of 1.2 (0.3–7.1) years for late infantile MLD and 3.7 (0.2–6.8) years for juvenile MLD [1]. During this time patients often experience a rapid deterioration and loss of skills. Our study showed that this is particularly evident in the earlier onset forms of MLD, where substantial irreversible damage occurs within a period of months. In a recent study of 97 patients with MLD, all patients with motor involvement exhibited rapid disease progression regardless of the subtype [20]. The rate of progression was greater when motor symptoms were present at disease onset. In late juvenile and adult-onset patients, the course of the disease was as rapid as in the early onset forms, when motor symptoms were present at disease onset [20]. Our study reported that patients with adult-onset MLD displayed cognitive and behavioural issues prior to loss of motor skills, in agreement with other studies [2, 10].

Parents and caregivers expressed their frustration that their early concerns were not always taken seriously, and many visited several specialists before appropriate testing was performed. For the majority in this study, early diagnosis prior to symptoms appearing was only achieved due to the diagnosis of MLD in an older sibling. The benefits of early detection reach far beyond the patient and early diagnosis would greatly reduce the emotional and mental toil on the family caused by the long and tumultuous diagnostic process [21]. Moreover, our data shows that the knowledge NBS would provide would allow parents to make informed reproductive decisions in the future. Most respondents also felt that the benefits of an early diagnosis, such as early treatment and the choice to be included in clinical trials, far outweighed the potential impacts of receiving a false positive screening result.

PKU was the first inherited condition to be screened for in the UK. If diagnosed shortly after birth, irreversible damage can be avoided by prescribing a phenylalanine-restricted diet [22]. The health benefits were clearly apparent and NBS for PKU was implemented in many countries worldwide [23]. Due to the success of PKU screening, and the availability of novel treatments, more inherited conditions have been added to NBS programmes over the years. Although NBS is available for six other inborn errors of metabolism in the UK, NBS for MLD disease is not included. 90% (n = 19) of respondents in our survey felt that NBS was extremely important, and 86% (n = 18) thought that it would have helped to inform their reproductive choices. A recent systematic review of 36 studies, of which 12 were from the UK, suggested that NBS was poorly understood and that the potential impact of receiving a positive result was not considered by parents. In fact, most parents were unaware screening had taken place [24]. For a disease to be part of an NBS programme, a set of ten screening criteria must be met [25]. These criteria have been in use since NBS began more than 50 years ago and have subsequently been modified due to advances in technology [26]. For NBS, “an accepted treatment for patients” is a criterion for diseases to be included. NBS would dramatically reduce the burden on patients, families and healthcare clinicians through the diagnosis period and recent modelling has demonstrated the cost-effectiveness of NBS in other inborn errors of metabolism [27].

Recent breakthroughs in potential treatments offer some hope to patients and their families but despite this progress, therapies are only beneficial in pre-symptomatic patients or those at very early stages of disease, emphasising the need for a rapid diagnosis [15]. Substantial progress in gene therapy provides much optimism for the treatment and management of MLD and its availability will offer patients and families a vastly improved quality of life. Libmeldy^™^ is the first gene therapy approved for eligible patients with early-onset MLD. Eligible patients are characterised by biallelic mutations in the *ARSA* gene leading to a reduction of ARSA enzymatic activity in children with late infantile or early juvenile subtypes, without clinical manifestations or with early clinical manifestations of the disease. Results demonstrated high levels of reconstituted ARSA activity in cerebrospinal fluid, arrested neurodegeneration, and a favourable safety profile [28]. Other treatments under investigation include intrathecal replacement of recombinant ARSA (Clinical trials identifier: NCT01303146), and AAV-mediated gene therapy, based on the direct multiple injection of ARSA expressing viral vectors into the brain of patients (Clinical trials identifier: NCT01801709) [2, 27, 29]. Although there are reasons for optimism, the need for early diagnosis is apparent. The advent of gene therapy and enzyme replacement therapy for the treatment of several rare diseases, such as MLD, have opened the door for their inclusion on NBS panels.

Qualitative research provides real-world insight from the perspective of the subject. Although our study was small, due to the rarity of MLD, important insights on first symptoms, disease progression and views on NBS were ascertained through open-ended questioning. This allowed issues to be explored in detail and new ones identified. The inherent nature of this methodology highlights potential limitations, such as the requirement for parents and caregivers to remember when first symptoms developed retrospectively. This descriptive study relied upon individual memory and was not able to validate findings against medical records. This may be particularly important when reporting diagnostic delay, as the timings of when symptoms appeared can be subjective and should therefore be considered as approximations only. Finally, some respondents left gaps in the online survey when reporting symptoms at different timepoints, which in turn led to some variability in the data available for each patient/respondent. Despite these limitations, the results of our study provide a strong case for MLD to be included in the UK NBS panel.

## Conclusion

Our data highlight the considerable delay from the appearance of first symptoms to MLD diagnosis and demonstrates the rapid deterioration of both motor and cognitive function during this time. The rapid rate of disease progression MLD makes it an essential candidate for NBS, particularly now as the first gene therapy has been approved.

## Data Availability

In order to maintain patient confidentiality, raw data from this study is not available.
